# High‐frequency repetitive transcranial magnetic stimulation protects against cerebral ischemia/reperfusion injury in rats: Involving the mitigation of ferroptosis and inflammation

**DOI:** 10.1002/brb3.2988

**Published:** 2023-04-16

**Authors:** Gui‐Juan Zhou, Dan‐Ni Liu, Xia‐Rong Huang, Qi Wu, Wei‐Bin Feng, Ya‐Hua Zeng, Hong‐Ya Liu, Jing Yu, Zi‐Jian Xiao, Jun Zhou

**Affiliations:** ^1^ The First Affiliated Hospital, Department of Rehabilitation, Hengyang Medical School University of South China Hengyang Hunan P. R. China; ^2^ The First Affiliated Hospital, Rehabilitation Laboratory, Hengyang Medical School University of South China Hengyang Hunan P. R. China; ^3^ The First Affiliated Hospital, Department of Neurology, Hengyang Medical School University of South China Hengyang Hunan P. R. China

**Keywords:** cerebral ischemia/reperfusion injury, ferroptosis, inflammation, repetitive transcranial magnetic stimulation

## Abstract

**Background and aim:**

Repetitive transcranial magnetic stimulation (rTMS) has been found to attenuate cerebral ischemia/reperfusion (I/R) injury. However, its effects and mechanism of action have not yet been clarified. It has been reported that cerebral I/R injury is closely associated not only with ferroptosis but also with inflammation. Hence, the current study aimed to investigate whether high‐frequency rTMS attenuates middle cerebral artery occlusion (MCAO)‐induced cerebral I/R injury and further to elucidate the mediatory role of ferroptosis and inflammation.

**Methods:**

The protective effects of rTMS on experimental cerebral I/R injury were investigated using transient MCAO model rats. Neurological scores and pathological changes of cerebral ischemic cortex were assessed to evaluate the effects of rTMS on cerebral I/R injury. The involvement of ferroptosis and that of inflammation were examined to investigate the mechanism underlying the effects of rTMS.

**Results:**

High‐frequency rTMS remarkably rescued the MCAO‐induced neurological deficits and morphological damage. rTMS treatment also increased the mRNA and protein expression of glutathione‐dependent peroxidase 4, decreased the mRNA and protein levels of acyl‐CoA synthetase long‐chain family member 4 and transferrin receptor in the cortex. Moreover, rTMS administration reduced the cerebrospinal fluid IL‐1β, IL‐6, and TNF‐α concentrations.

**Conclusion:**

These findings implicated that high‐frequency rTMS alleviates MCAO‐induced cerebral I/R injury, and the underlying mechanism could involve the inhibition of ferroptosis and inflammation. Our study identifies rTMS as a promising therapeutic agent for the treatment of cerebral I/R injury. Moreover, the mechanistic insights into ferroptosis and inflammation advance our understanding of it as a potential therapeutic target for diseases beyond cerebral ischemia stroke.

## Graphical abstract

High‐frequency rTMS rescues the MCAO‐induced neurological deficits and alleviates MCAO‐induced cerebral I/R injury, probably by inhibiting ferroptosis and inflammation.

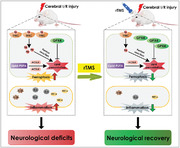

## 1 INTRODUCTION

Cerebral ischemia stroke is one of the prime causes of mortality and disability globally (Johnson et al., 2019). It has been widely accepted that recanalization of occluded cerebral vessels through intravenous thrombolysis and endovascular treatment is necessary and fruitful to salvage damaged neuronal cells in the acute treatment time window (Boers et al., 2019; Lin et al., 2019). However, recanalization therapy can lead to cerebral ischemia/reperfusion (I/R) injury (Sun et al., 2018). Although reperfusion is essential for reducing infarct size and substantially salvages neuronal cells, it is linked with the oxidative stress‐related damage and inflammatory reactions in cells that survive following the initial ischemic injury (Eltzschig & Eckle, 2011). These latter events trigger the cascade of cerebral I/R injury and result in extensive tissue damage, neuronal death, and irreversible neurological consequences (White et al., 2000), which severely limit the applications of vascular recanalization in the therapy for ischemic stroke. To this point, there is an urgent need for innovative and effective strategies for the treatment of cerebral I/R injury.

Repetitive transcranial magnetic stimulation (rTMS) is a novel neuromodulatory technique to noninvasively modulate the brain activity and function through magnetic pulses (Castrillon et al., 2020), and its role in protecting against ischemic cerebrovascular disease has been universally supported (Le et al., 2014; Luo et al., 2017; Yozbatiran et al., 2009). Clinical studies have confirmed that rTMS promotes the recovery of motor dysfunctions (Harvey et al., 2018), chronic pain (Khedr et al., 2005), dysphagia (Verin & Leroi, 2009), and aphasia (Barwood et al., 2012) in patients with ischemic stroke. Additionally, rTMS attenuates the neurological deficits which evaluated by the neurological function scores in rats with cerebral ischemia reperfusion (Hong et al., 2020). Although rTMS has been applied extensively in the clinic, the mechanisms through which rTMS exerts this profound efficacy have remained incompletely understood (Chervyakov et al., 2015).

Ferroptosis, a novel programmed cell death pathway driven by iron‐dependent lipid peroxidation, has been triggered by multiple mechanisms, including upregulation of intracellular labile iron levels, peroxidation of polyunsaturated fatty acids, and disturbance of glutathione (GSH) homeostasis (Stockwell et al., 2017). It has been reported that an increased body iron stores correlates with poor outcome after thrombolytic treatment in patients with acute ischemic stroke (Millan et al., 2007). In addition, iron chelation reduces I/R‐induced brain damage (Palmer et al., 1994). Of note, the inhibition of ferroptosis by ferrostatin‐1 alleviates cerebral I/R injury in mice (Alim et al., 2019; Tuo et al., 2017). These studies suggest that ferroptosis plays a crucial role in the pathological process of cerebral I/R injury. Thus, considering the protective effect of rTMS on cerebral I/R injury, in this work, we sought to investigate whether ferroptosis mediates the beneficial effect of rTMS in cerebral I/R injury rats.

In response to stress or stimuli, the innate immune system may respond by cell survival or death (Bolivar et al., 2019). The danger‐associated molecular patterns released from the cytoplasm during non‐apoptotic forms of cell death may act as signals to trigger sterile inflammation (Fuchs & Steller, 2015). Hence, cell death is a dynamic process more than a consequence of inflammation, and the process is closely related to the development of inflammatory diseases. I/R injury underlies the progress of sterile inflammation in stroke, and the major culprit is a dramatic burst of free radicals during the reperfusion phase after the ischemic period (Esposito et al., 2019). Depletion of GSH, increased lipid peroxidation products, as well as disorders of iron metabolism are common features in ferroptosis and inflammatory diseases (Mao et al., 2020), suggesting that ferroptosis may be involved in the progression of sterile inflammations. Indeed, it has been shown that the inhibition of ferroptosis downregulates the TLR4/TRIF/type I IFN signaling pathway to reduce sterile inflammation in heart I/R injury (Li et al., 2019). Additionally, accumulative studies reported that rTMS exerts its beneficial effects via its antioxidant action (Medina‐Fernandez et al., 2018). Therefore, we set out to explore the involvement of inflammation in rTMS‐mediated effects to interpret the possible underlying mechanism.

In the present work, we hypothesized that rTMS could mitigate ferroptosis and inhibit inflammation in cerebral I/R injury rats model to promote functional recovery. To examine this hypothesis, high‐frequency rTMS at frequency of 10 Hz was delivered to normal rats or rats subjected to middle cerebral artery occlusion (MCAO). Then the neurological function and histology were investigated. The 10 Hz rTMS administration could attenuate I/R‐induced neurological deficits and the loss of cortical neuron. The effects of 10 Hz rTMS on ferroptosis‐related indicators and inflammatory factors were systematical assessed. To our knowledge, this is the first study to investigate the effects of rTMS on ferroptosis after cerebral I/R injury.

## 2 MATERIALS AND METHODS

### 2.1 Animals and reagents

Male Sprague–Dawley rats (weighing 405–450 g; 12‐week) were purchased from Hunan SJA Laboratory Animal Center (Changsha, Hunan, China). All rats were housed under standard conditions: 12 h light/dark cycle, room temperature 23–27°C, humidity 45%–55%, and access to standardized laboratory pellet food and water. Adaptive feeding was conducted for 1 week before the treatment. All experiments were performed in compliance with the Regulations for the Administration of Affairs Concerning Experimental Animals (China) and approved by the Animal Use and Protection Committee of University of South China. Animal suffering was minimized to the greatest extent possible. Anti‐acyl‐CoA synthetase long‐chain family member 4 (ACSL4) antibody (*Catalog no*. A16848) and anti‐transferrin receptor (TFRC) antibody (*Catalog no*. A5865) were obtained from ABclonal (Wuhan, Hubei, China), and Anti‐glutathione‐dependent peroxidase 4 (GPX4) antibody (*Catalog no. ab125066*) was purchased from Abcam (Cambridge, UK). Anti‐β‐actin antibody (*Catalog no. 66009‐1‐Ig*), HRP‐conjugated Affinipure Goat Anti‐Mouse IgG (H + L) (*Catalog no*. SA00001‐1), and HRP‐conjugated Affinipure Goat Anti‐Rabbit IgG (H + L) (*Catalog no*. SA00001‐2) were supplied by Proteintech (Chicago, IL, USA).

### 2.2 MCAO (transient middle cerebral artery occlusion) rat model and grouping

With some slight modifications (Wang et al., 2008) from Zea Longa's previous method (Longa et al., 1989), the transient MCAO operation using the intraluminal filament method was performed. Briefly, a peritoneal injection of 1% sodium pentobarbital (Sigma, St. Louis, MO, USA) (0.5 mL per 100 g) was used to anesthetize the rats. Subsequently, supine rats were incised on the neck midline, and the left common carotid artery, the left external carotid artery and the left inner carotid artery (ICA) exposed. Then a monofilament nylon suture with a rounded tip (diameter of 0.43 mm) (L4300, Guangzhou Jialing Biotechnology, Guangzhou, Guangdong, China) was carefully inserted into the left ICA and occluded the middle cerebral artery. After 1.5 h of ischemia, the nylon suture was withdrawn gently to achieve reperfusion. Sham rats were treated with the same procedures without inserting the nylon suture. Throughout the procedures, the whole body temperature was maintained at 37.0 ± 0.5°C with a heating pad and monitored using a rectal temperature probe.

The rats were randomly assigned to three experimental groups: the sham group (sham group), the MCAO ischemia‐reperfusion model group (MCAO group), and the rTMS treatment group (MCAO + rTMS group). All the rats, except those in sham group, were subjected to MCAO procedure, and only the rats in the MCAO + rTMS group underwent rTMS treatment.

### 2.3 Repetitive transcranial magnetic stimulation (rTMS) treatment

rTMS was delivered using a magnetic‐electric stimulator (YRD‐CCY‐1, Wuhan, Hubei, China) with a round coil (Y064, 3.5 cm diameter). The treatment was initiated at 6 h after reperfusion. All procedures were based on published animal experiment protocols in previous studies (Guo et al., 2017). In brief, the coil was positioned perpendicular to the cortex in the cortical surface projection area of the left cerebral hemisphere of rats. The treatment protocol consisted of stimulation for 10 s followed by rest for 50 s (repeated 10 times) at a rate of 10 Hz. The stimulation intensity was set at 33% of the maximum stimulator output. These steps were repeated daily for 7 days.

### 2.4 Assessment of neurological function

The neurological deficits were assessed using the Longa scoring system (Longa et al., 1989) 7 days after reperfusion, and score of neurological function was recorded. The scoring criteria were as follows: 0, no observable deficit; 1, failure to extend right forepaw on lifting the whole body by tail; 2, circling to the contralateral side; 3, loss of walking or righting reflex; 4, no spontaneous motor activity.

### 2.5 Hematoxylin–eosin (HE) staining

The pathological changes of brain tissues were assessed using hematoxylin–eosin (HE) staining. Freshly obtained brain samples were fixed overnight in 4% formaldehyde and embedded in paraffin. Then HE staining was performed according to the instruction manual of the HE staining kit (G1120, Solarbio, Beijing, China). The observations were made under the microscope.

### 2.6 Reverse transcription quantitative PCR (RT‐qPCR)

The relative mRNA expression was analyzed by reverse transcription quantitative PCR (RT‐qPCR) as previously described (Hu et al., 2016). The total RNA extract was performed with TRIzol Plus RNA Purification Kit (15596026, Thermo Scientific, MA, USA) as directed by the manufacturer, and the concentration of total RNA was measured spectrophotometrically. RNA was reverse‐transcribed into cDNA using a reverse transcription kit (CW2569, CWBIO, Beijing, China). Subsequently, the quantitative PCR was performed using the UltraSYBR Mixture (CW2601, CWBIO, Beijing, China) according to the manufacturer's instructions. Amplification conditions were 95°C for 10 min, followed by 40 cycles of 95°C for 15 s and 60°C for 30 s. The primers used in PCR amplification and real‐time RT‐PCR for specific genes are listed in Table 1. The relative amount of mRNA was calculated using the comparative *Ct* (ΔΔ*Ct*) method. All specific genes mRNA expression were normalized against β‐actin.

**TABLE 1:** Reverse transcription quantitative PCR (RT‐qPCR) primers used in this study

Gene	Primer sequence	
	Forward	Reverse
β‐Actin	ACATCCGTAAAGACCTCTATGCC	TACTCCTGCTTGCTGATCCAC
GPX4	AATTCGCAGCCAAGGACATCG	ATTCGTAAACCACACTCGGCGTA
ASCL4	CCCTGTCCCGTTCTCATACCG	CAAACACCAAAAGGCAACAAGCA
TFRC	GCCACAAGCCAAACAATATCCG	ACACTGCTCCCGATAATGTGA

Abbreviations: GPX4, glutathione‐dependent peroxidase 4; TFRC, transferrin receptor.

### 2.7 Western blotting analysis

Protein expressions of GPX4, ASCL4, and TFRC were assessed by western blotting analysis according to the standard protocol. After euthanizing the rats via anesthetic overdose, left cerebral cortical tissues were immediately harvested to homogenize with RIPA buffer on the ice. Proteins were extracted from the supernatant and quantified with BCA Protein Assay Kit (Beyotime, Shanghai, China). Then total proteins were separated by 10%–12% sodium dodecyl sulfate‐polyacrylamide gel under denaturing conditions and transferred to polyvinylidene difluoride membranes (Merck Millipore Ltd., USA). After being blocked with 5% nonfat milk, the membranes were incubated with relevant primary antibodies (rabbit anti‐GPX4 (1:5000), rabbit anti‐ASCL4 (1:2000), mouse anti‐TFRC (1:2000), mouse anti‐β‐tubulin (1:5000)) overnight at 4°C. On the following day, the membranes were washed followed by incubation with secondary antibodies (HRP‐conjugated Affinipure Goat Anti‐Mouse IgG (H + L) (1:5000) and HRP‐conjugated Affinipure Goat Anti‐Rabbit IgG (H + L) (1:5000)) for 2 h (room temperature). Target proteins were visualized on the membranes using an enhanced chemiluminescence kit (Millipore Corporation, Billerica, MA, USA). Intensities of target protein bands, normalized to β‐actin, were quantified using ImageJ software (NIH, Bethesda, MD, USA).

### 2.8 Enzyme‐linked immunosorbent assay (ELISA)

The concentrations of IL‐1β, IL‐6, and TNF‐α were detected using the following commercially available enzyme‐linked immunosorbent assay (ELISA) kits: IL‐1β, Rat Interleukin 1β (IL‐1β) ELISA Kit (CUSABIO, Wuhan, Hubei, China); IL‐6, Rat Interleukin 6 (IL‐6) ELISA Kit (CUSABIO); and TNF‐α, Rat Tumor necrosis factor α ELISA Kit (CUSABIO). The concentration of each inflammatory cytokine in the cerebrospinal fluid (CSF) of rats was determined by ELISA according to the manufacturer's instructions.

### 2.9 Statistical analysis

Statistical analysis was performed using SPSS 26.0 (SPSS Inc., Chicago, IL, USA). Data are presented as mean ± S.E.M., except for the neurological scores. Data of neurological scores were analyzed by nonparametric tests (Kruskal–Wallis test) followed by a Dunn's post hoc test. For other data that met normal distribution assumptions and equal variances, one‐way analyses of variance were used with *LSD* post hoc tests. *p* Values <.05 were considered to indicate statistical significance in all statistical tests.

## 3 RESULTS

### 3.1 High‐frequency rTMS attenuates cerebral I/R injury in MCAO‐treated rats

**FIGURE 1:**
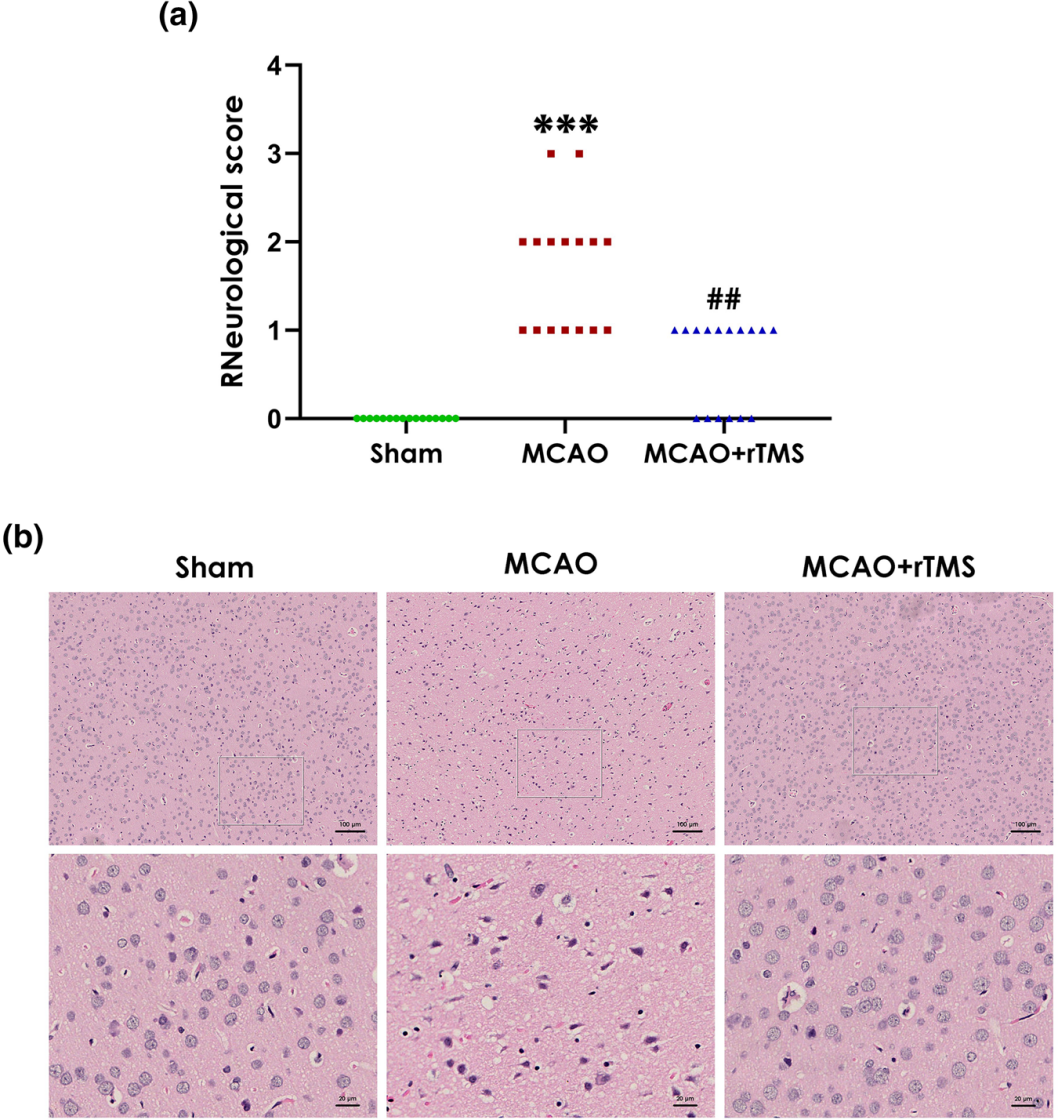
Effects of high‐frequency repetitive transcranial magnetic stimulation (rTMS) on the neurological function and histomorphology of cerebral ischemic cortex in middle cerebral artery occlusion (MCAO)‐treated rats. Six hours after MCAO operation, the rats were treated with high‐frequency rTMS (10 Hz, once daily for 7 days). (A) The neurological scores. (B) Representative image of hematoxylin–eosin (HE)‐stained sections from a rat brain cerebral cortex (upper panel: original magnification, scale bar, 100 μm; lower panel: magnification of the framed regions in upper panel, scale bar, 20 μm), (*n* = 3). Values are median with upper and lower quartiles (*n* = 16). ^***^ *p* < .001, versus sham group; ^##^ *p* < .01, versus MCAO group.

To investigate whether high‐frequency rTMS alleviates the neurological impairment, we used the Longa scoring system to assess neurological function of rats after MCAO. The results revealed that administration of high‐frequency rTMS attenuated MCAO‐induced increase in the neurological score (Figure 1A). In general, a higher score indicates decreased neurological function. So these data indicated that high‐frequency rTMS attenuates the neurological deficits of MCAO‐treated rats.

To further confirm the neuroprotective effect of rTMS, brain slices were stained with HE to assess the morphological changes. As shown in Figure 1B, HE staining evidenced a disorderly arrangement with cytoplasmic looseness, abnormal neuronal swelling, karyopyknosis, and cellular vacuolation in the MCAO group as compared with the sham group. Of note, such histological damage in the cortical ischemic zone could be significantly reversed following treatment with rTMS, implicating that high‐frequency rTMS improves morphological damage in the cortical ischemic area of MCAO‐treated rats. Taken together, these data demonstrated that high‐frequency rTMS alleviates the cerebral I/R injury in MCAO‐treated rats.

### 3.2 High‐frequency rTMS attenuates ferroptosis in the ischemic cortex of MCAO‐treated rats

**FIGURE 2:**
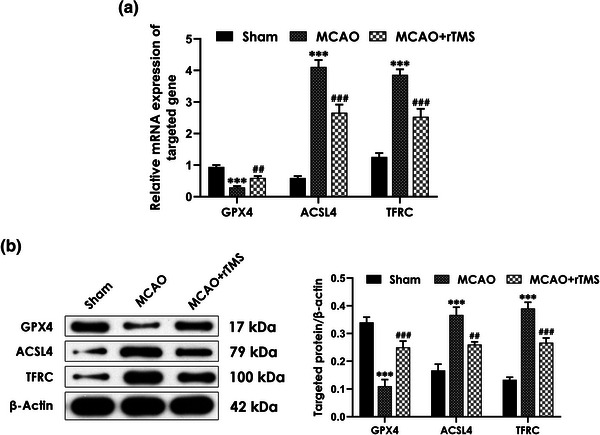
Effect of high‐frequency repetitive transcranial magnetic stimulation (rTMS) on cortical expression of genes related to ferroptosis in middle cerebral artery occlusion (MCAO)‐treated rats. Rats were treated with high‐frequency rTMS (10 Hz, once daily for 7 days) after MCAO operation. After behavioral experiments completed, (A) the mRNA expression of glutathione‐dependent peroxidase 4 (GPX4), ASCL4, and transferrin receptor (TFRC) as well as (B) the protein expression of GPX4, ASCL4, and TFRC in the cortex were detected by reverse transcription quantitative PCR (RT‐qPCR) and western blotting analysis, respectively. Values are means ± S.E.M., (*n* = 6). ^***^ *p* < .001, versus sham group; ^##^ *p* < .01, ^###^ *p* < .001, versus MCAO group.

It has been reported that excessive ferroptosis has been linked to cerebral I/R injury (Li et al., 2021). To determine the involvement of ferroptosis in rats treated with MCAO, and whether rTMS‐induced rescuing effects are associated with alleviating ferroptosis, the mRNA and protein expression of genes related to ferroptosis were assessed. The RT‐qPCR results showed that MCAO significantly increased the mRNA expression levels of ACSL4 and TFRC in the cortex but decreased the mRNA expression level of GPX4, which were reversed by rTMS treatment (Figure 2A). Consistently, MCAO‐treated rats exhibited significant increases in the protein expression levels of ASCL4 and TFRC as well as decrease in the protein expression level of GPX4, which were also significantly abolished by treatment with rTMS (Figure 2B). These data demonstrated that high‐frequency rTMS alleviates in the ischemic cortex of MCAO‐treated rats.

### 3.3 High‐frequency rTMS inhibits inflammation in the CSF of MCAO‐treated rats

**FIGURE 3:**
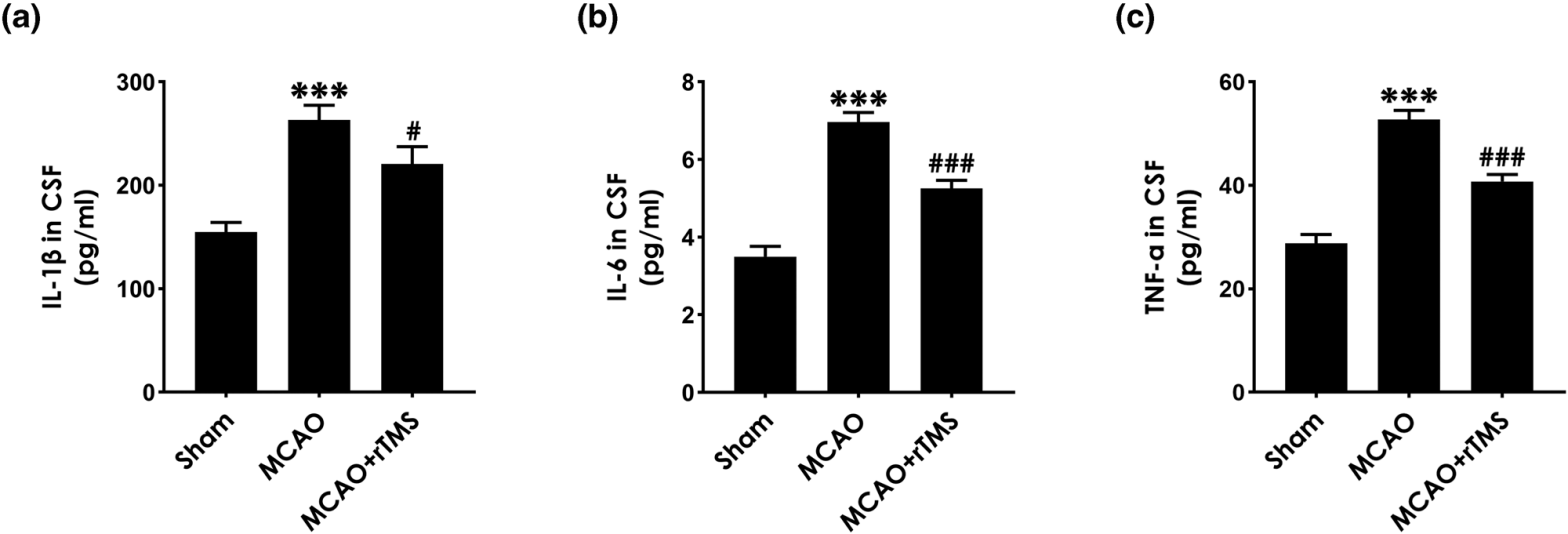
Effects of high‐frequency repetitive transcranial magnetic stimulation (rTMS) on the levels of proinflammatory cytokines (IL‐1β, IL‐6, and TNF‐α) in the cerebrospinal fluid (CSF) of middle cerebral artery occlusion (MCAO)‐treated rats. Six hours after MCAO operation, the rats were treated with high‐frequency rTMS (10 Hz, once daily for 7 days). After behavioral experiments completed, the CSF of rats was collected, and the concentrations of IL‐1β (A), IL‐6 (B), and TNF‐α (C) in the CSF were measured by using Elisa. Values are means ± S.E.M., (*n* = 8). ^***^ *p* < .001, versus sham group; ^#^ *p* < .05, ^###^ *p* < .001, versus MCAO group.

To investigate whether rTMS‐exerted neuroprotective effects in MCAO‐exposed rats are associated with inhibiting inflammation, inflammatory cytokines, including IL‐1β, IL‐6, and TNF‐α in CSF measured by ELISA test. As shown in Figure 3, the concentrations of IL‐1β (Figure 3A), IL‐6 (Figure 3B), and TNF‐α (Figure 3C) in the CSF of MCAO‐exposed rats were markedly upregulated, which significantly reduced by treatment with high‐frequency rTMS. These results implicated that high‐frequency rTMS attenuates the inflammation in the CSF of MCAO‐treated rats.

## 4 DISCUSSION

The present study set out to determine the protective effects of high‐frequency rTMS on MCAO‐induced cerebral I/R injury. rTMS improved the neurological scores and the histomorphology of cortical ischemic area in MCAO model rats. rTMS also rescued the MCAO‐changed mRNA and protein expression of genes related to ferroptosis. Furthermore, rTMS treatment reduced the CSF concentrations of IL‐1β, IL‐6, and TNF‐α. These findings suggested that high‐frequency rTMS attenuates cerebral I/R injury induced by MCAO, and the mechanism of this protective action involves the inhibition of ferroptosis and inflammation.

The transient MCAO is a classic model of cerebral I/R injury, which most closely simulates human ischemic stroke (Gong et al., 2017). Rats with transient MCAO undergo focal cerebral ischemia reperfusion, exhibiting neurological motor deficits and histopathological alterations. In this study, increased neurological deficit scores and damaged morphology in cortex were observed in MCAO‐treated rats, indicating that the rat model of cerebral I/R injury was successfully established. As a novel noninvasive approach with long‐lasting effects, rTMS has been increasingly used in stroke rehabilitation in recent years (Harvey et al., 2018; Verin & Leroi, 2009). There is also evidence that different frequencies of rTMS exerts neuroprotective effects on ischemic stroke in different ischemic stroke rodent models (Guo et al., 2017; Hong et al., 2020; Luo et al., 2017). In our study, 10 Hz high‐frequency rTMS alleviated MCAO‐induced neurological deficit and morphological damage, indicating that high‐frequency rTMS improved cerebral I/R injury in MCAO model rats. More importantly, our findings may shed light on the development of a novel therapeutic application for cerebral I/R injury.

Ferroptosis represents a novel programmed cell death type that is iron dependent and associated with lipid oxidative metabolism controlled by GPX4 (Stockwell, 2022). In recent years, a growing number of evidence has emphasized the role of ferroptosis in ischemic stroke (Cui et al., 2021; Li et al., 2021). The expression of GPX4 was significantly decreased (Fu et al., 2022), whereas levels of ASCL4 (Chen et al., 2021) and TFRC1 (Ding et al., 2011) were greatly increased in animal MCAO model, consistent with our results. It is well established that ferroptosis could be triggered by inhibiting the expression and/or activity of GPX4 (Kremer et al., 2021), whose depletion results in overwhelming lipid peroxidation and cell death. Furthermore, ACSL4 is an essential component for ferroptosis execution, which dictates ferroptosis sensitivity and is regarded as a ferroptosis biomarker (Doll et al., 2017). In addition, TFRC transports transferrin‐bound iron from the extracellular environment into cytoplasm, contributing to the cellular iron pool required for ferroptosis (Feng et al., 2020). Strikingly, in our present study, treatment with high‐frequency rTMS reversed the reduced GPX4 level as well as the elevated ASCL4 and TFRC levels in the cortex of MCAO rats, indicating that rTMS may inhibit ferroptosis in MCAO‐treated rats. Therefore, we elucidate that rTMS‐attenuated ferroptosis contributes to its protective role against MCAO‐induced cerebral I/R injury.

Increasing attention has been paid to proinflammatory mediators and their role in cerebral I/R injury (Franke et al., 2021). Immediately after ischemic stroke, immune cells in the central nervous system are excessively activated, leading to the release of several inflammatory factors, such as IL‐1β, IL‐6, and TNF‐α. Then these inflammatory cytokines can further result in severe inflammatory responses and aggravate brain injury (Liu et al., 2020). Inhibiting the release of inflammatory factors during reperfusion could significantly mitigate this brain injury (Franke et al., 2021). In the present study, we investigated the effect of rTMS on the neuroinflammation after MCAO insult. We found that rTMS administration significantly reduced the levels of proinflammatory cytokines (IL‐1β, IL‐6, and TNF‐α) in the CSF after MCAO challenge, indicating that rTMS inhibits neuroinflammation in MCAO‐treated rats. Taken together, these results implicate that improving role of rTMS in the neurological function of MCAO‐exposed rats is mediated by the suppression of inflammation. However, some limitation must be acknowledged. Future studies are warranted to validate the effects of rTMS on ferroptosis and inflammation using agonists or inhibitors. Furthermore, clinical studies should be conducted to evaluate the application of this technique in the treatment of cerebral ischemia.

In conclusion, we demonstrated that high‐frequency rTMS alleviated cerebral I/R injury, the underlying mechanisms probably involve the mitigation of ferroptosis and inflammation. From a clinical translational point of view, our findings provide a rationale for clinical application of rTMS in ischemic stroke. Furthermore, targeting the pathways that regulate ferroptosis might represent a novel preventive therapeutic strategy to alleviate ischemic stroke. More importantly, anti‐inflammation strategies hold great promise for the treatment of cerebral I/R injury.

## CONFLICT OF INTEREST STATEMENT

All authors have declared no competing financial interests and no conflict of interests to the presented work.

### PEER REVIEW

The peer review history for this article is available at https://publons.com/publon/10.1002/brb3.2988.

## Data Availability

The data that support the findings of this study are available from the corresponding author upon reasonable request.
